# Real‐world first‐line treatment with pembrolizumab for non‐small cell lung carcinoma with high PD‐L1 expression: Updated analysis

**DOI:** 10.1002/cam4.70036

**Published:** 2024-07-18

**Authors:** Yasuyuki Ikezawa, Ryo Morita, Hidenori Mizugaki, Kazunari Tateishi, Keiki Yokoo, Toshiyuki Sumi, Hajime Kikuchi, Yasuo Kitamura, Atsushi Nakamura, Maki Kobayashi, Mari Aso, Nozomu Kimura, Fumiaki Yoshiike, Furuta Megumi, Hisashi Tanaka, Motoki Sekikawa, Tsutomu Hachiya, Keiichi Nakamura, Fumihiro Hommura, Noriaki Sukoh, Kenichiro Ito, Takashi Kikuchi, Toshihiko Agatsuma, Hiroshi Yokouchi

**Affiliations:** ^1^ Department of Respiratory Medicine Oji General Hospital Tomakomai Japan; ^2^ Department of Respiratory Medicine, Faculty of Medicine Hokkaido University Sapporo Japan; ^3^ Department of Respiratory Medicine Akita Kousei Medical Center Akita Japan; ^4^ Department of Respiratory Medicine National Hospital Organization Hokkaido Cancer Center Sapporo Japan; ^5^ Department of Advanced Medical Development The Cancer Institute Hospital of Japanese Foundation for Cancer Research Tokyo Japan; ^6^ First Department of Internal Medicine Shinshu University School of Medicine Matsumoto Japan; ^7^ Department of Respiratory Medicine Teine Keijinkai Hospital Sapporo Japan; ^8^ Department of Respiratory Medicine Hakodate Goryoukaku Hospital Hakodate Japan; ^9^ Department of Respiratory Medicine Obihiro‐Kousei General Hospital Obihiro Japan; ^10^ Department of Respiratory Medicine Kushiro City General Hospital Kushiro Japan; ^11^ Department of Pulmonary Medicine Sendai Kousei General Hospital Sendai Japan; ^12^ Department of Respiratory Medicine Miyagi Cancer Center Natori Japan; ^13^ Department of Respiratory Medicine Yamagata Prefectural Central Hospital Yamagata Japan; ^14^ Department of Respiratory Medicine Tohoku University Graduate School of Medicine Sendai Japan; ^15^ Department of Respiratory Medicine Nagano Municipal Hospital Nagano Japan; ^16^ Department of Respiratory Medicine Hirosaki University, Graduate School of Medicine Hirosaki Japan; ^17^ Department of Respiratory Medicine Steel Memorial Muroran Hospital Muroran Japan; ^18^ Department of Respiratory Medicine Japanese Red Cross Suwa Hospital Suwa Japan; ^19^ Department of Respiratory Medicine National Hospital Organization Asahikawa Medical Center Asahikawa Japan; ^20^ Department of Respiratory Medicine Sapporo City General Hospital Sapporo Japan; ^21^ Department of Respiratory Medicine National Hospital Organization Hokkaido Medical Center Sapporo Japan; ^22^ Department of Respiratory Medicine KKR Sapporo Medical Center Sapporo Japan; ^23^ Department of Respiratory Medicine Iwate Prefecture Isawa Hospital Oshu Japan; ^24^ Department of Respiratory Medicine Shinshu Ueda Medical Center Ueda Japan

**Keywords:** chemotherapy, clinical cancer research, lung cancer, non‐small cell lung cancer

## Abstract

**Background:**

Selecting pembrolizumab monotherapy (MONO) or pembrolizumab plus platinum‐based chemotherapy (COMB) for patients with nonsmall cell lung cancer (NSCLC) and high programmed death‐ligand 1 (PD‐L1) expression is an important issue in clinical practice. We previously conducted a retrospective multicenter observational study of patients with NSCLC and high PD‐L1 expression who received MONO or COMB as a first‐line treatment. Here, we report updated data and evaluate the long‐term outcomes.

**Methods:**

We performed a retrospective multicenter study of 298 patients with NSCLC and high PD‐L1 expression who received MONO or COMB as first‐line treatment between December 2018 and January 2020. We reviewed the medical records and assessed the clinical efficacy and toxicity using a prolonged data cutoff.

**Results:**

In total, 164 (median age: 74 years) and 134 (median age: 68 years) patients received MONO and COMB, respectively; patients who received COMB were younger and had better performance statuses (0–1). At the prolonged data cutoff, the median follow‐up was 20.2 (range: 0.1–41.4) months. The median progression‐free survivals were 7.5 and 13.1 months, and overall survivals (OSs) were 17.2 and 33.7 months for MONO and COMB, respectively. Treatment discontinuation rates were 21.9% and 20.1% for the MONO and COMB, respectively. With prolonged follow‐up, although COMB demonstrated an OS benefit and higher objective response rate than MONO, in the propensity score matching analysis COMB didn't demonstrate a significant benefit compared to the MONO.

**Conclusions:**

COMB may be effective as a first‐line treatment for NSCLC with high PD‐L1 expression in a selected subset of patients.

## 1 INTRODUCTION

The efficacy of immune checkpoint inhibitors (ICIs) has recently been reported, emerging as a standard therapy for patients with advanced‐stage nonsmall cell lung cancer (NSCLC). In particular, when compared with platinum‐based chemotherapy, patients with high programmed death‐ligand 1 (PD‐L1) expression (PD‐L1 tumor proportion score [TPS] ≥50%) who received first‐line treatment with pembrolizumab (a programmed cell death protein‐1 [PD‐1] inhibitor and humanized monoclonal antibody) as either monotherapy (MONO) or combined with chemotherapy (COMB) demonstrated improved progression‐free survival (PFS) and overall survival (OS).^1^, ^2^, ^3^, ^4^, ^5^ Conversely, a lower incidence of treatment‐related adverse events (AEs) were reported in patients who received MONO compared with COMB. These results suggest that MONO and COMB are beneficial first‐line treatments for advanced NSCLC with high PD‐L1 expression, and that MONO is better tolerated than COMB.

Based on the limited data, selection between MONO and COMB for patients with NSCLC with high PD‐L1 expression in the real world is important. We previously conducted a retrospective multicenter observational trial, Hokkaido Lung Cancer Clinical Study Group (HOT)/North Japan Lung Cancer Study Group (NJLCG) 2001 (UMIN000040223), with a data cutoff date of August 31, 2020.^6^ The median PFSs were 7.1 months and 13.1 months in the MONO and COMB groups, respectively, which was significantly different. Additionally, the objective response rates (ORRs) were 41% and 67.4% in the MONO and COMB groups, respectively. The incidence of AEs of grade 3 or higher, or associated with treatment discontinuation, was similar in both groups. Our results suggested that COMB may be a promising first‐line treatment for NSCLC with high PD‐L1 expression and a good performance status (PS). Furthermore, other retrospective studies have reported real‐world outcomes of pembrolizumab (or other ICIs) alone or in combination with platinum‐based chemotherapy for advanced NSCLC with high PD‐L1 expression.^7^, ^8^, ^9^ However, these findings remain controversial, and it is unclear whether MONO or COMB is superior for patients with NSCLC and high PD‐L1 expression in the real world.

Herein, we report the updated PFS, OS, ORR, 2‐year survival rate, and safety data (additional 17‐month follow‐up for a median follow‐up duration of 20.2 months) of 298 patients with NSCLC and high PD‐L1 expression, who received MONO or COMB as a first‐line treatment. We aimed to evaluate the long‐term outcomes of patients in this setting, as well as conduct statistical analyses to support the selection of appropriate treatments in the real world.

## 2 MATERIALS AND METHODS

### 2.1 Study Design

The retrospective, multicenter, observational study (HOT/NJLCG2001) design has been previously described [6]. Eligible patients had previously untreated advanced NSCLC with a PD‐L1 TPS of ≥50%, and no sensitizing *EGFR, ALK*, or *ROS‐1* alterations; additionally, they received MONO or COMB as the first‐line treatment between December 2018 and January 2020. The efficacies and toxicities of MONO and COMB were also evaluated. We reviewed the medical records of 34 institutions of HOT, NJLCG, and Shinshu area institutions in Japan. This study was approved by the institutional review boards of all the institutions, which waived the need for informed consent owing to the anonymous nature of the data. The extended data cutoff date for this study was January 31, 2022.

### 2.2 Data collection

We assessed patient characteristics, therapeutic regimens, treatment periods, PFS, OS, 2‐year survival rate, and AEs; age, sex, smoking status, histology, clinical stage, PD‐L1 status, and Eastern Cooperative Oncology Group (ECOG) PS score at the start of initial treatment were recorded. We also assessed PD‐L1 expression, and recorded the therapeutic regimens (MONO or COMB) and type of chemotherapy (carboplatin + pemetrexed, cisplatin + pemetrexed, carboplatin + nab‐paclitaxel, and carboplatin + paclitaxel). We extracted AE types of grade 3 or higher, and AE types leading to treatment discontinuation using common terminology criteria for adverse events (CTCAE ver. 5.0).^10^ Tumor response was measured using the Response Evaluation Criteria in Solid tumors version 1.1 (RECIST 1.1),^11^ and assessments were performed at each participating institution.

### 2.3 Statistical analysis

Details were previously described.^6^ PFS was defined as the time interval between the initial treatment administration and disease progression or death; patients without documented clinical or radiographic disease progression or those who were still alive were censored on the date of the last follow‐up. OS was defined as the time interval between initial treatment administration and any cause of death. PFS and OS were evaluated using the Kaplan–Meier method and compared using a two‐sided log‐rank test. To reduce selection bias and obtain similar comparison groups, we used a propensity score‐matched pair method combined with covariate adjustment, with age and PS as adjustment factors, to analyze patients treated with MONO or COMB. Hazard ratios (HRs) and 95% confidence intervals (CIs) were estimated using the Cox proportional hazards regression model. All *p*‐values were two‐sided, and the threshold for statistical significance was set at *p* < 0.05. All statistical analyses were performed using JMP Ver. 13.2.0 (SAS Institute Inc., Cary, NC, USA).

## 3 RESULTS

### 3.1 Patient characteristics

**FIGURE 1:**
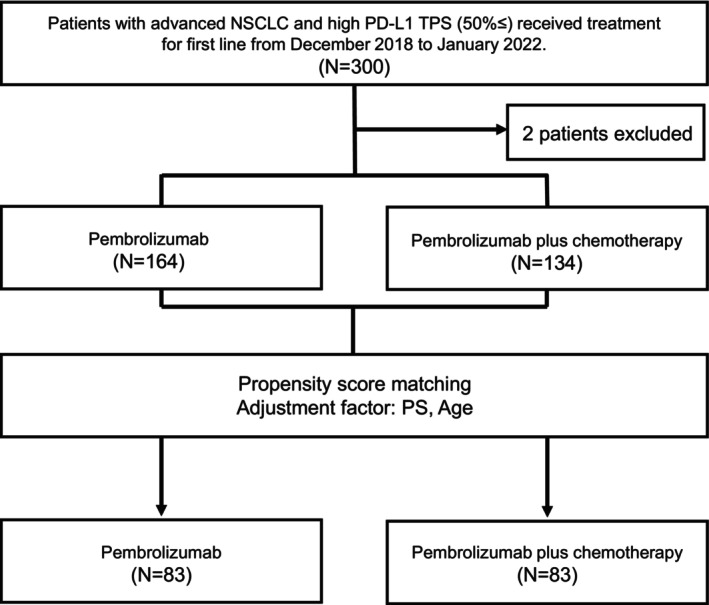
As shown in the flowchart, 298 patients received either pembrolizumab monotherapy or pembrolizumab plus platinum‐based chemotherapy between December 2018 and January 2020. We used the propensity score‐matched pairing method, and 1:1 matching yielded 83 patient pairs.

At the data cutoff date (August 31, 2020), a total of 300 patients with advanced NSCLC and a TPS ≥50% who underwent first‐line treatment were enrolled in this study; two were excluded at the prolonged data cutoff date (January 31, 2022). Of the 298 patients, 164 (55%) and 134 (45%) received MONO and COMB, respectively (Figure 1). The baseline patient characteristics are summarized in Table 1. The median patient ages were 74 (range: 52–89) years and 68 (range: 45–84) years in the MONO and COMB groups, respectively. Patients in the COMB group were younger and had a better PS (0–1) (*p* < 0.01) (Table 1).

**TABLE 1:** Baseline and treatment characteristics.

Characteristics	No (%)			*p* Value
	All	MONO	COMB	
	(*n* = 298)	(*n* = 164)	(*n* = 134)	
Age years				
Median (Range)	71 (45–89)	74 (52–89)	68 (45–84)	<0.01
Sex				
Male	236 (79.3)	129 (78.6)	107 (79.9)	0.99
Female	62 (20.7)	35 (21.4)	27 (20.1)	
Smoking status				
Current smoker	71 (23.7)	31 (18.9)	40 (29.6)	0.11
Former smoker	186 (62.5)	108 (65.9)	78 (58.5)	
Never Smoker	41 (13.8)	25 (15.2)	16 (11.9)	
Histology				
Adenocarcinoma	170 (57.0)	91 (55.4)	79 (59.0)	0.57
Squamous cell carcinoma	83 (27.9)	46 (28.1)	37 (27.6)	
Others	45 (15.1)	27 (16.5)	18 (13.4)	
Stage				
III	44 (14.8)	30 (18.3)	14 (10.4)	0.03
IV	215 (72.2)	108 (65.9)	107 (79.9)	
Recurrence	39 (13.0)	26 (15.8)	13 (9.7)	
Performance Status				
0	92 (30.5)	33 (20.1)	59 (44.0)	<0.01
1	148 (50.0)	83 (50.6)	65 (48.5)	
2	41 (13.8)	33 (20.1)	8 (6.0)	
3	17 (5.7)	15 (9.2)	2 (1.5)	
PD‐L1 status				
50%–74%	95 (31.9)	55 (33.5)	40 (29.9)	0.62^a^
75%–89%	83 (27.8)	45 (27.5)	38 (28.4)	
≥90%	99 (33.2)	60 (36.6)	39 (29.1)	
≥50% (details are unknown)	21 (7.1)	4 (2.4)	17 (12.6)	
Regimens				
CDDP+PEM + Pembrolizumab	—	—	35 (26.1)	
CBDCA+PEM + Pembrolizumab	—	—	47 (35.1)	
CBDCA+nab‐PTX + Pembrolizumab	—	—	49 (36.6)	
CBDCA+PTX + Pembrolizumab	—	—	3 (2.2)	

^a^ Excluding cases whose PD‐L1 status details are unknown.

Abbreviations: CBDCA, Carboplatin; CDDP, Cisplatin; nab‐PTX, nab‐Paclitaxel; PEM, Pemetrexed; PTX, Paclitaxel.

### 3.2 Efficacy

**FIGURE 2:**
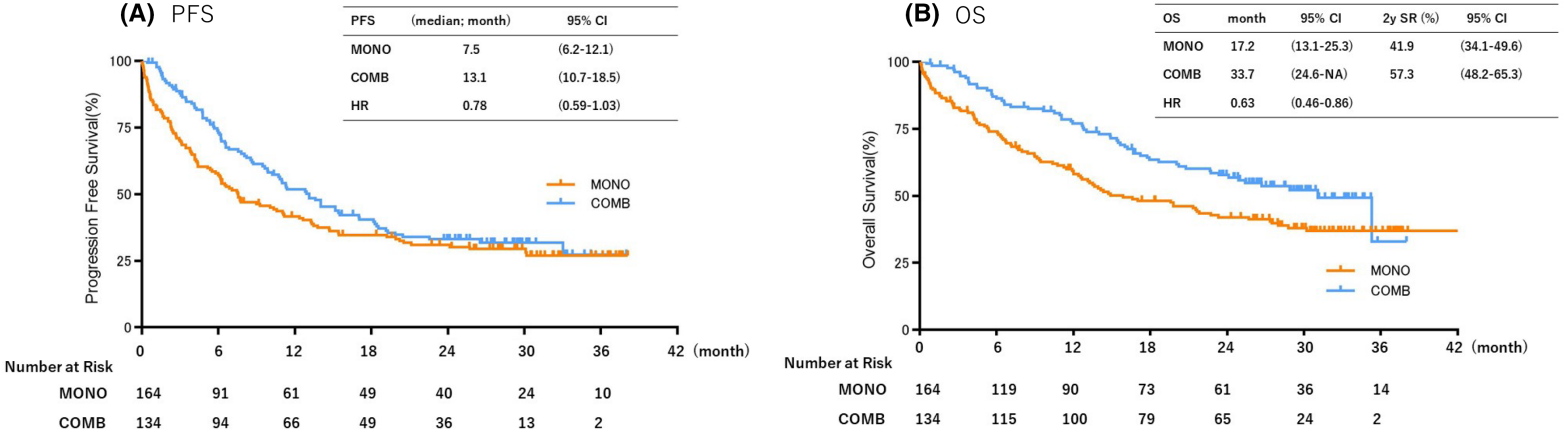
Kaplan–Meier curves of (A) progression‐free survival and (B) overall survival of all patients receiving pembrolizumab monotherapy (MONO) or pembrolizumab plus platinum‐based chemotherapy (COMB). mo, month; OS, overall survival; PFS, progression‐free survival.

At the prolonged data cutoff date (January 31, 2022), the median follow‐up was 20.2 months (range: 0.1–41.4). The median PFSs were 7.5 months (95% CI: 6.2–12.1) and 13.1 months (95% CI:10.7–18.5) in the MONO and COMB groups, respectively (HR: 0.78; 95% CI: 0.59–1.03; *p* Value = 0.076) (Figure 2A). The median OS was 17.2 months (95% CI: 13.1–25.3) in the MONO group and 33.7 months (95% CI: 24.6– not reached [NR]) in the COMB group (HR: 0.63; 95% CI: 0.46–0.86; *p* Value = 0.045). The 2‐year survival rates were 41.9% (95% CI: 34.1–49.6) and 57.3% (95% CI: 48.2–65.3) in the MONO and COMB groups, respectively. (Figure 2B). This analysis revealed an OS benefit for COMB in the total patient population.

**FIGURE 3:**
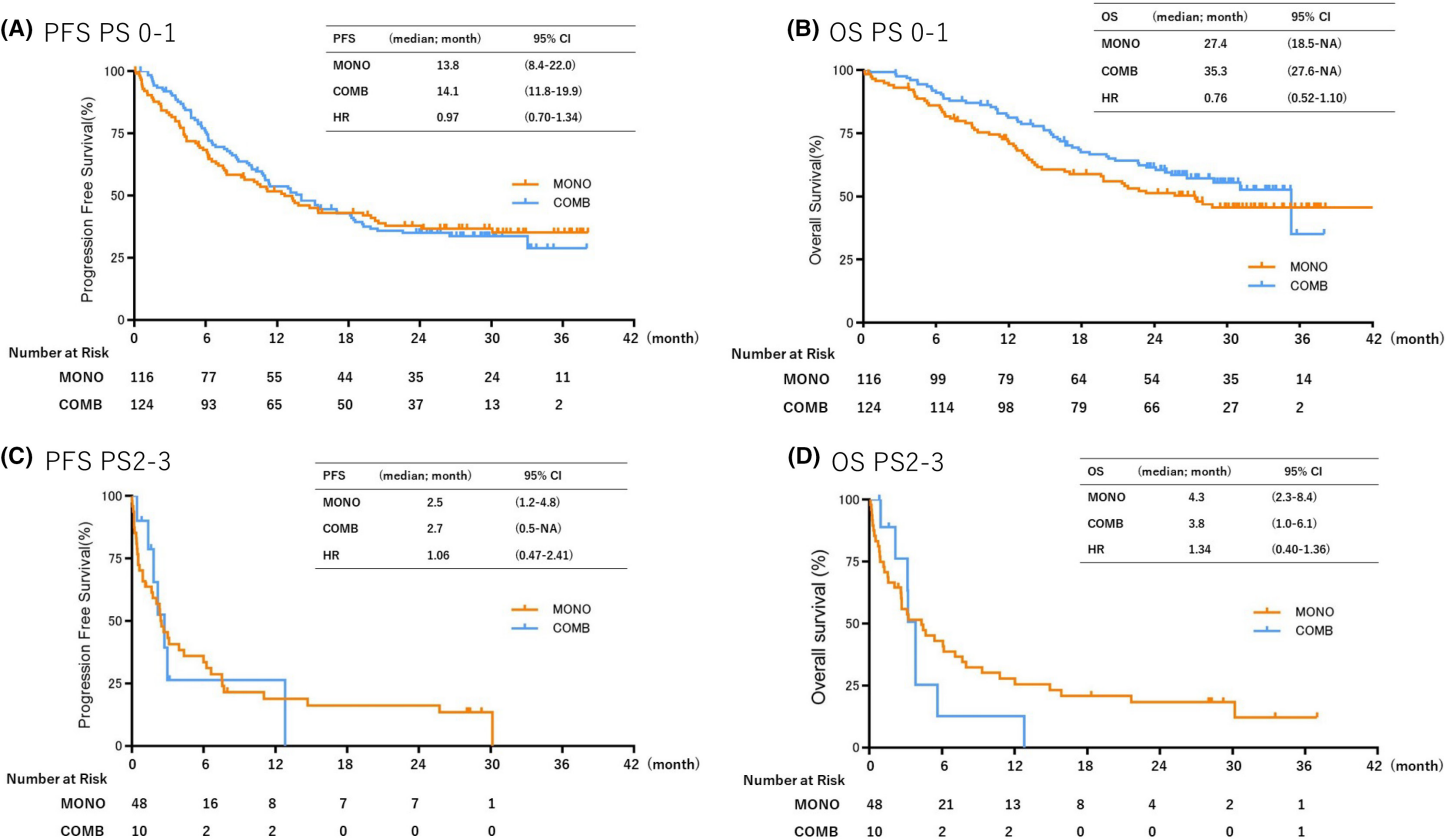
Kaplan–Meier curves of progression‐free survival (PFS) and overall survival (OS) of all patients receiving pembrolizumab monotherapy (MONO) or pembrolizumab plus platinum‐based chemotherapy (COMB) according to ECOG performance status. (A) PFS with PS 0–1, (B) PFS with PS ≥2, (C) OS with PS 0–1, (D) OS with PS ≥2. ECOG, Eastern Cooperative Oncology Group; mo, Month; PS, Performance status.

**FIGURE 4:**
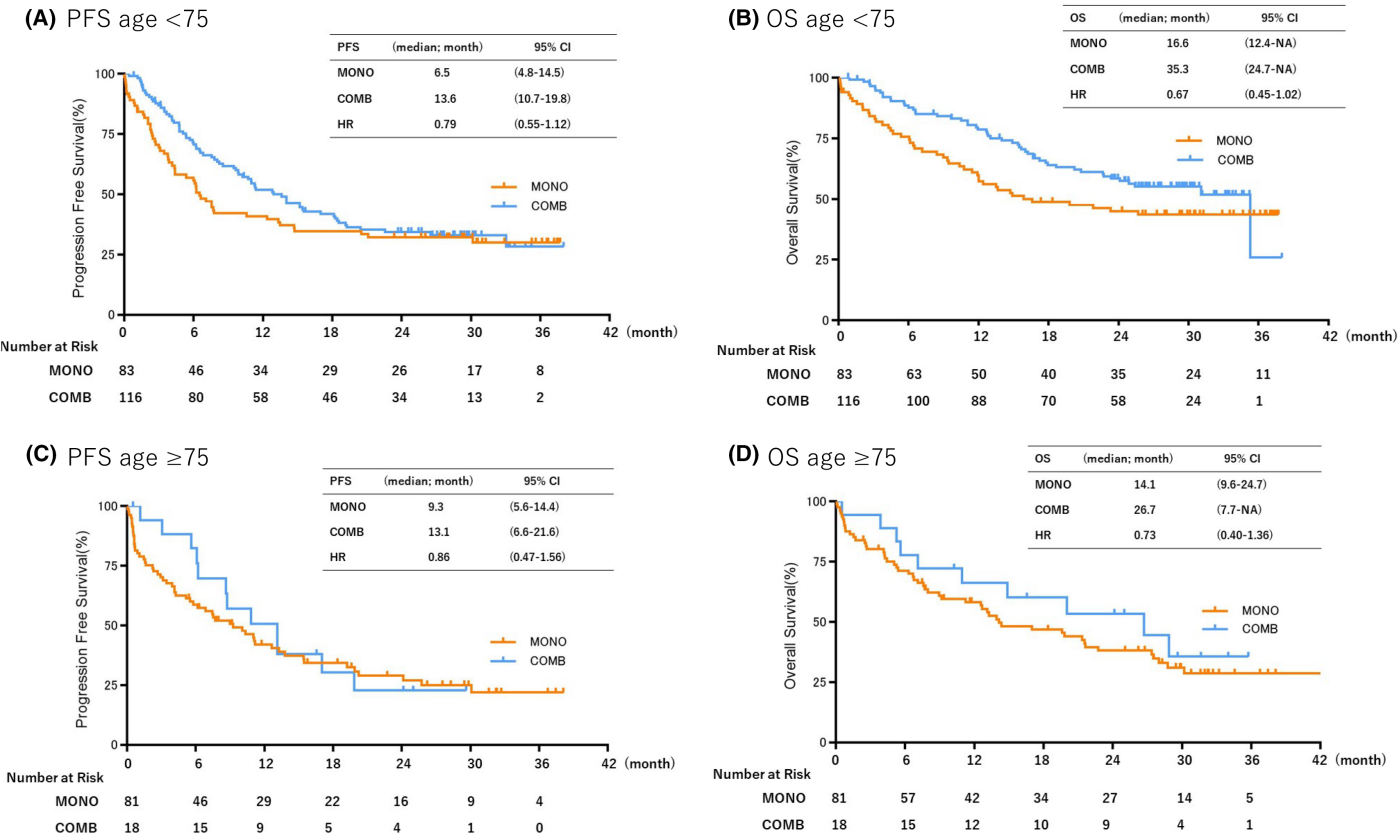
Kaplan–Meier curves of progression‐free survival (PS) and overall survival (OS) of patients receiving pembrolizumab monotherapy (MONO) or pembrolizumab plus platinum‐based chemotherapy (COMB) according to age. (A) PFS of patients aged <75 years, (B) PFS of patients aged ≥75 years, (C) OS of patients aged <75 years, (D) OS of patients aged ≥75 years. mo, Month.

Next, we evaluated PFS and OS in selected PS and age subgroups. In the PS subgroup analysis, there were no statistically significant differences in PFS or OS between MONO and COMB treatments in the PS 0–1 subgroup (Figure 3A, B). However, the OS tended to be shorter with COMB than MONO in the PS 2–3 subgroup (PFS: 2.5 vs. 2.7 months, respectively [HR: 1.06; 95% CI: 0.47–2.41; *p* Value = 0.88]; OS: 4.3 vs. 3.8 months, respectively [HR: 1.34; 95% CI: 0.4–1.36; *p* Value = 0.38]) (Figure 3C). In the age subgroups, the younger group (<75 years old) showed a trend toward better outcomes than the older group; however, there were no statistically significant differences in the median PFS and OS between COMB and MONO (PFS: 6.5 vs. 13.6 months, respectively [HR: 0.79; 95%CI: 0.55–1.12; *p* Value = 0.21]; OS:16.6 vs. 35.3 months, respectively [HR 0.67; 95% CI: 0.45–1.02; *p* Value = 0.051]) (Figure 4).

A confirmed objective response occurred in 69 patients (42.1%) treated with MONO (complete response [CR], *n* = 6; partial response [PR], *n* = 63) and 92 patients (68.7%) treated with COMB (CR, *n* = 4; PR, *n* = 88) (Table 2). Additionally, the progressive disease rates were 28.7% (47/166) in the MONO group and 11.1% (15/134) in the COMB group (Table 2).

**TABLE 2:** Best tumor response to first line pembrolizumab monotherapy or pembrolizumab plus platinum‐based chemotherapy.

	No. (%)			
	MONO (*n* = 164)		COMB (*n* = 134)	
Tumor response				
ORR	69	(42.1)	92	(68.7)
DCR	107	(65.2)	118	(88.1)
Best overall response				
CR	6	(3.7)	4	(3.0)
PR	63	(38.4)	88	(65.7)
SD	38	(23.1)	26	(19.4)
PD	47	(28.7)	15	(11.1)
NE	10	(6.1)	1	(0.8)

Abbreviations: COMB, pembrolizumab plus platinum‐based chemotherapy; CR, complete response; DCR, disease control rate; MONO, pembrolizumab monotherapy; NE, not be evaluated; ORR, objective response rate; PD, progressive disease; PR, partial response; SD, stable disease.

### 3.3 Safety and toxicity

At the prolonged data cutoff date, 223 patients (74.8%) discontinued treatment. The treatment discontinuation rates were 82.9% (136/164) and 64.9% (87/134) in the MONO and COMB groups, respectively. Disease progression was the most common reason for treatment discontinuation; 40 (24.4%) and 34 (25.3%) patients in the MONO and COMB groups, respectively, discontinued treatment due to AEs. The AEs leading to discontinuation did not differ significantly between the MONO and COMB groups. The most frequently reported AE was pneumonitis, reported in 16 patients (9.6%) receiving MONO and 18 patients (13.4%) receiving COMB; all patients with pneumonitis discontinued treatment, including 12 patients (7.2%) in the MONO group and 11 (8.2%) in the COMB group with grade 3 pneumonitis (Table 3). No treatment‐related deaths occurred in either group.

**TABLE 3:** Baseline and treatment characteristics after propensity score matching.

Characteristics	No (%)						*p* Value
	All (*n* = 166)		MONO (*n* = 83)		COMB (*n* = 83)		
Age years							
Median (Range)	70	(49–88)	70	(53–84)	70	(49–84)	0.847
Sex							
Male	130	(78.3)	65	(77.2)	65	(77.2)	>0.99
Female	36	(21.7)	18	(22.8)	18	(22.8)	
Smoking status							
Current smoker	41	(24.7)	22	(26.5)	19	(22.9)	0.723
Former smoker	104	(62.7)	52	(62.7)	52	(62.7)	
Never Smoker	21	(12.6)	9	(10.8)	12	(14.4)	
Histology							
Adenocarcinoma	90	(54.2)	45	(54.2)	45	(54.2)	0.49
Squamous cell carcinoma	49	(29.5)	27	(32.5)	22	(26.5)	
Others	27	(16.3)	11	(13.3)	16	(19.3)	
Stage							
III	28	(16.9)	13	(15.7)	15	(18.0)	0.554
IV	116	(69.9)	61	(73.5)	55	(66.3)	
Recurrence	22	(13.2)	9	(10.8)	13	(15.7)	
Performance Status							
0	57	(34.3)	28	(33.7)	29	(34.9)	0.998
1	91	(54.8)	46	(55.4)	45	(54.2)	
2	14	(8.4)	7	(8.4)	7	(8.4)	
3	4	(2.5)	2	(2.4)	2	(2.4)	
PD‐L1 status							
50%–74%	55	(33.1)	28	(33.7)	27	(32.5)	0.304
75%–89%	48	(28.9)	25	(30.1)	23	(27.7)	
≥90%	57	(34.3)	25	(30.1)	32	(38.6)	
≥50% (details are unknown)	6	(3.7)	5	(6.1)	1	(1.2)	

### 3.4 Propensity score matching analysis

**FIGURE 5:**
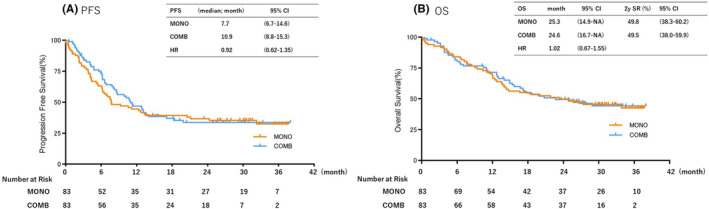
Kaplan–Meier curves of (A) progression‐free survival and (B) overall survival of patients receiving pembrolizumab monotherapy (MONO) or pembrolizumab plus platinum‐based chemotherapy (COMB) after propensity score matching. mo, month; OS, overall survival; PFS, progression‐free survival.

We used propensity score matching to adjust for factors that differed between the groups or were clinically important. Propensity score matching allowed the MONO and COMB scores to be almost identical regarding age and PS. To control for unbalanced conditions at the baseline between the two groups, 22 patients were excluded owing to a lack of detailed PD‐L1 assessments. We used a propensity score‐matched pairing method with age and PS as adjustment factors; 1:1 matching yielded matched pairs of 83 patients in both groups (Figure 1 and Table 4). In the matched cohort, the median PFS was 7.7 months (95% CI: 6.7–14.6) in the MONO group, and 10.9 months (95% CI: 8.8–15.3) in the COMB group (HR: 0.92, 95% CI: 0.62–1.35; *p* Value = 0.65) (Figure 5A). The median OS was 25.3 months (95% CI: 14.9–[NA]) in the MONO group and 24.6 months (95% CI: 16.7–[NA]) in the COMB group (HR: 1.02, 95% CI: 0.67–1.55; *p* Value = 0.93). The 2‐year survival rate was 49.8% (95% CI: 38.3–60.2) in the MONO group and 49.5% (95% CI: 38.0–59.9) in the COMB group (Figure 5B).

**TABLE 4:** Treatment‐related adverse events (AEs) ≧grade 3 and leading to the discontinuation of all treatment.

AEs	No. (%)			
	MONO (*n* = 164)		COMB (*n* = 134)	
	≥G3	DISCON	≥G3	DISCON
Total	44 (26.8)	40 (24.4)	42 (31.3)	28 (26.7)
Pneumonitis	12 (7.2)	16 (9.6)	11 (8.2)	18 (13.4)
Rash	3 (1.8)	3 (1.8)	5 (3.7)	5 (3.7)
Hepatic dysfunction	3 (1.8)	2 (1.2)	4 (3.0)	3 (2.2)
Thromboembolic event	3 (1.8)	2 (1.2)	—	—
Adrenal insufficiency	4 (2.4)	2 (1.2)	1 (0.8)	1 (0.8)
Cholangitis	2 (1.2)	2 (1.2)	—	—
Enterocolitis	2 (1.2)	—	—	—
Neutrophil count decreased	1 (0.6)	1 (0.6)	8 (6.0)	—
Bronchiolitis	1 (0.6)	1 (0.6)	—	—
Fever	1 (0.6)	1 (0.6)	—	—
Kidney infection	1 (0.6)	1 (0.6)	—	—
Myelodysplastic syndrome	1 (0.6)	1 (0.6)	—	—
Nervous system disorder	1 (0.6)	1 (0.6)	—	—
Pericarditis	1 (0.6)	1 (0.6)	—	—
Pharyngeal ulcer	1 (0.6)	1 (0.6)	—	—
Uveitis	1 (0.6)	1 (0.6)	—	—
Hyponatremia	2 (1.2)	—	1 (0.8)	—
Biliary obstruction	1 (0.6)	—	—	—
Polymyalgia rheumatica	1 (0.6)	—	—	—
Vasculitis	1 (0.6)	—	—	—
Diarrhea	—	2 (1.2)	—	—
Eyelid function disorder	—	1 (0.6)	—	—
Lung infection	—	1 (0.6)	—	—
Intestinal perforation	—	—	3 (2.2)	—
Renal dysfunction	—	—	3 (2.2)	3 (2.2)
Anemia	—	—	2 (1.5)	4 (3.0)
Heart failure	—	—	1 (0.8)	1 (0.8)
Anorexia	—	—	1 (0.8)	—
Peritonitis	—	—	1 (0.8)	—
Platelet count decreased	—	—	1 (0.8)	—
Thyroid gland malfunction	—	—	1 (0.8)	—
Edema	—	—	—	1 (0.8)

Abbreviations: COMB, pembrolizumab plus platinum‐based chemotherapy; DISCON, discontinuation; MONO, pembrolizumab monotherapy.

## 4 DISCUSSION

In this updated analysis, COMB continued to demonstrate an OS benefit and better ORR than MONO as a first‐line therapy for advanced NSCLC with high PD‐L1 expression. Discontinuation rates due to AEs were similar in both groups. However, there were no statistically significant differences in median OS between the COMB and MONO groups in the PSM analysis. Based on our results, although the COMB group did not show a statistically significant superiority compared to the MONO group in PSM, some patients may derive benefits from COMB depending on patient background factors such as PS and age.

Our study showed that COMB was associated with a longer OS and higher ORR than MONO (67.9% vs. 42.2%), and our results are consistent with those of several prospective studies and meta‐analyses [1–4, 12]. In another retrospective study, Chen et al.^7^ reported that the PFS and OS of MONO and COMB were 9.6 and 12.4 (*p* Value <0.001), and 28.9 and NA (*p* Value = 0.005), respectively. By contrast, Dudhik et al.^8^ observed no significant differences in long‐term outcomes between MONO and COMB. In the real world, because large biases were observed in baseline characteristics such as age and PS, which are often significantly related to the prognosis of patients, these results may be controversial. Additionally, at the prolonged data cutoff date, we evaluated the OS in subgroups by PS and age; in clinical practice, these are relevant to elucidate the treatment options for patients with a PS ≥2 who are aged ≥75 years. In the subgroup analysis of PS, although there was no significant difference, the OS tended to be better with COMB than with MONO in the PS 0–1 group. In the age subgroups, the younger group (<75 years) showed a trend toward a better median OS with COMB than with MONO. Based on these results, background factors such as PS and age are important when selecting MONO or COMB, even in patients with high PD‐L1 expression.

In the extended follow‐up analysis, the proportion of patients experiencing AEs of grade 3 or higher, and AEs associated with treatment discontinuation, were similar; however, the incidence of AEs increased in both groups when compared with our previous report.^6^ Tang et al.^13^ reported the pattern of time‐to‐onset of AEs caused by PD‐1/PD‐L1 inhibitors. The pooled median time to onset of AEs of grade 3 or higher ranged from 14.1 weeks to 123.4 weeks for PD‐1/PD‐L1 inhibitors, and was significantly longer than that of all AEs. Although AEs generally occur within several months after the initiation of ICIs, they may appear several months or even years after completing treatment. Our updated analysis and this report suggest that the occurrence pattern and onset of AEs vary; thus, we should pay attention to AEs after the initiation and even completion of treatment.

PSM analysis demonstrated that the effect of chemotherapy on immunotherapy prolonged PFS by approximately 12 months, and reduced the rate of early exacerbation in COMB. Alternatively, immunotherapy may contribute to the long‐term survival of patients with NSCLC. OS and 2‐year survival rates were also similar after PSM in both groups. These results are consistent with those of previous reports,^12^, ^13^, ^14^, ^15^ suggesting that although the effect of immunotherapy on chemotherapy may be limited for patients with advanced NSCLC and high PD‐L1 expression, COMB treatment should be considered as an option in cases of good PS.

This study had some limitations; first, this was a retrospective study, which may have led to a bias in regimen selection. However, we used PSM to reduce bias and compare the treatment efficacies of MONO and COMB. Second, the sample size was relatively small; however, because our data were similar to that of the meta‐analysis,^12^ our sample size reflects real‐world data. Third, not all adverse events were recorded. Last, although we extended the observation period to obtain in‐depth data on the long‐term efficacy and safety, the duration was not sufficient.

## 5 CONCLUSIONS

COMB continued to demonstrate an OS benefit and a higher ORR compared with MONO at longer follow‐up but did not show a significant advantage over the MONO group in PSM analysis. Based on this extended real‐world cohort, COMB continued to demonstrate an OS benefit and a higher ORR compared to MONO during longer follow‐up. However, in PSM analysis, COMB did not show a significant advantage over the MONO group. Depending on patient characteristics such as age and PS, COMB may be effective as a first‐line treatment for NSCLC with high PD‐L1 expression in a selected subset of patients.

## AUTHOR CONTRIBUTIONS

**Yasuyuki Ikezawa:** Conceptualization (lead); data curation (equal); formal analysis (equal); investigation (equal); writing – original draft (lead). **Ryo Morita:** Conceptualization (equal); data curation (equal); formal analysis (lead); investigation (equal); writing – original draft (lead). **Hidenori Mizugaki:** Conceptualization (lead); data curation (equal); formal analysis (equal); supervision (lead); writing – review and editing (lead). **Kazunari Tateishi:** Conceptualization (equal); data curation (equal); formal analysis (equal); investigation (equal); writing – review and editing (equal). **Keiki Yokoo:** Conceptualization (equal); data curation (equal); formal analysis (equal); investigation (equal); writing – review and editing (equal). **Toshiyuki Sumi:** Investigation (equal); writing – review and editing (supporting). **Hajime Kikuchi:** Investigation (equal); writing – review and editing (supporting). **Yasuo Kitamura:** Investigation (equal); writing – review and editing (supporting). **Atsushi Nakamura:** Investigation (equal); writing – review and editing (supporting). **Maki Kobayashi:** Investigation (equal); writing – review and editing (supporting). **Mari Aso:** Investigation (equal); writing – review and editing (supporting). **Nozomu Kimura:** Investigation (equal); writing – review and editing (supporting). **Fumiaki Yoshiike:** Investigation (equal); writing – review and editing (supporting). **Furuta Megumi:** Investigation (equal); writing – review and editing (supporting). **Hisashi Tanaka:** Investigation (equal); writing – review and editing (supporting). **Motoki Sekikawa:** Investigation (equal); writing – review and editing (supporting). **Tsutomu Hachiya:** Investigation (equal); writing – review and editing (supporting). **Keiichi Nakamura:** Investigation (equal); writing – review and editing (supporting). **Fumihiro Hommura:** Investigation (equal); writing – review and editing (supporting). **Noriaki Sukoh:** Investigation (equal); writing – review and editing (supporting). **Kenichiro Ito:** Investigation (equal); writing – review and editing (supporting). **Takashi Kikuchi:** Investigation (equal); writing – review and editing (supporting). **Toshihiko Agatsuma:** Investigation (equal); writing – review and editing (supporting). **Hiroshi Yokouchi:** Investigation (equal); writing – review and editing (supporting).

## FUNDING INFORMATION

This research did not receive any specific grant from funding agencies in the public, commercial, or not‐for‐profit sectors.

## CONFLICT OF INTEREST STATEMENT

The authors have no conflict of interest.

## ETHICS STATEMENT

This study was conducted in accordance with the Declaration of Helsinki and was approved by the Institutional Review Board of Oji General Hospital.

## CONSENT

Given the retrospective nature of the study, the requirement for informed consent was waived.

## CLINICAL TRIAL REGISTRATION

This study was registered at UMIN‐clinical trial registration (UMIN000040223).

## CONSENT

Given the retrospective nature of the study, the requirement for informed consent was waived.

## Data Availability

The data obtained this study and used to generate these findings will be made available, by the corresponding author, for reasonable request.
